# Significant Within-Individual Variability in VCTE Liver Stiffness Measurements at Two Intercostal Spaces in Subjects with MASLD: Implications for Evaluating Improvement in Liver Fibrosis After Weight-Loss or Liver-Directed Therapy

**DOI:** 10.3390/diseases12110288

**Published:** 2024-11-09

**Authors:** Jordan S. Woodard, Jena Velji-Ibrahim, Gary A. Abrams

**Affiliations:** 1 Department of Medicine, University of Kentucky Healthcare, Lexington, KY 40536, USA; jordan.woodard2@uky.edu; 2 Prisma Health—Upstate, Department of Medicine, School of Medicine-Greenville, University of South Carolina, Greenville, SC 29605, USA; jena.velji-ibrahim@prismahealth.org

**Keywords:** MASLD, obesity, elastography, fibrosis, resmetirom

## Abstract

Introduction: Studies have compared the group-averages of liver stiffness measures (LSMs) from multiple rib spaces by vibration-controlled transient elastography (VCTE) to stage liver fibrosis. No previous study has assessed within-individual liver stiffness variation from two rib spaces in individuals with metabolic-dysfunction associated steatotic liver disease (MASLD). Methods: We evaluated within-individual LSM variation according to body weight classification and its clinical implication. From October 2019 to March 2024, VCTE was performed on MASLD patients or those at high risk, in accordance with FibroScan guidelines. The LSMs were categorized into stages: <5 kPa (stage 0), 5–7.99 kPa (stage 1), 8–9.99 kPa (stage 2), 10–13.99 kPa (stage 3), and 14+ kPa (stage 4). Measurements with 10 values and IQR/median ≤ 0.30 were included, using SPSS V25.0 for analysis. Results: Among 1107 subjects (age 54.4 ± 13.9 years, 56.9% female), 7.7% were normal weight, 20.7% overweight, 28.9% class 1 obesity, 21.3% class 2 obesity, and 21.2% class 3 obesity. Significant within-individual variation was noted: 67% (0–2 kPa) variation, 23.4% (2.1–6 kPa), and 10% (≥6.1 kPa). Class 3 obese individuals had the maximum variation. Comparing the group-average of LSM at each ICS site showed that 95% of individuals were within one fibrosis stage. Conclusions: While LSM group-averages at different rib sites provides reliable fibrosis staging, significant within-individual variability exists especially in class 3 obesity. This should be considered when serial LSM assessments are used to assess medical therapeutic efficacy.

## 1. Introduction

The classification of chronic liver disease has evolved, introducing the term steatotic liver disease (SLD), which encompasses metabolic-dysfunction associated steatotic liver disease (MASLD) [1]. MASLD is the most prevalent category of SLD and affects over 30% of the global population [2]. In adults, MASLD comprises a combination of fatty liver identified on imaging (abdominal US/CT/MRI), with at least one of the following metabolic risk factors or on medication: BMI ≥ 25 kg/m^2^ or a waist circumference > 94 cm (male) or 80 cm (female), fasting glucose 100 mg/dL or HbA1 c ≥ 5.7% or type 2 diabetes, blood pressure ≥ 130/85, triglycerides ≥ 150 mg/dL, or HDL ≤ 40 mg/dL (men) or ≤50 mg/dL (women) [3]. Individuals taking medications for the above risk factors would apply despite normalization on treatment. Individuals with MASLD that also drink alcohol are divided into two categories, MASLD (average daily amounts of alcohol men up to 60 g (men) or 50 g (women), and MetALD for subjects who consume more alcohol.

An increased recognition of MASLD has led to an improved fibrosis screening algorithm. In individuals at low risk for MASLD, characterized by the absence of type 2 diabetes mellitus (T2DM) or two or more metabolic risk factors, initial screening consists of a fibrosis-4 (FIB-4) calculation that includes routine serology (AST, ALT, platelets) and age, with subsequent testing every two years if <1.3 (or <2.0 if older than 65 years) [3]. Those with an elevated FIB-4 value, or individuals deemed high-risk as mentioned above, are encouraged to undergo a non-invasive test (NIT) to assess for significant or advanced fibrosis. The most commonly used NITs include vibration-controlled transient elastography (VCTE, Fibro Scan), magnetic resonance elastography (MRE), or serologic tests such as the enhanced liver fibrosis (ELF) test.

VCTE transmits a sheer wave from the probe into the liver, and a transducer on the probe calculates both velocity and attenuation to provide liver stiffness measurements (LSM) and controlled-attenuation parameter score (CAP) as a surrogate for staging liver fibrosis and grading steatosis, respectively [4,5]. The operator uses real-time ultrasound feedback to identify an adequate space in the right lobe of the liver, obtaining 10 measurements from a single intercostal space (ICS), typically between the eighth through eleventh ICS sites in the midaxillary line [5]. The 10 LSM measurements should have an IQR/median ≤ 30% to be considered accurate values.

Although VCTE is widely used due to its accessibility and affordability compared to MRE, its effectiveness lies predominantly in ruling out advanced fibrosis, but with a low positive predictive value for ruling in advanced fibrosis. Previous sensitivity and specificity have been reported as 0.83 and 0.89 for cirrhosis and 0.79 and 0.78 for significant fibrosis (F2) [6]. VCTE measurements will become increasingly utilized to identify MASLD subjects with stages 2 or 3 fibrosis that qualify for the recently FDA-approved liver-directed medication (Resmetirom) and to determine its effectiveness in the resolution of liver fibrosis by performing serial LSM measurements. Despite the widespread use of VCTE, previous studies evaluating multiple ICS sites were limited to small sample sizes and primarily non-obese subjects with viral hepatitis [7,8,9,10]. To the best of our knowledge, no study has focused on the within-individual variability of LSM values (kilopascals, kPa) obtained from two different ICS sites across BMI classes. We aimed to calculate the range of LSM individual variation across all body mass index (BMI) classes and potential clinical implications.

## 2. Materials and Methods

### 2.1. Study Design and Patient Selection

Participants were identified through hepatology referral for VCTE evaluation of confirmed or suspected MASLD (elevated liver enzymes, obesity, T2DM, or metabolic syndrome) between October 2019 and March 2024.

Basic demographic data was collected for all patients, including age, gender, race, height, weight, and body mass index (BMI), as well as data on comorbidities including T2DM, hypertension, and dyslipidemia. Additionally, laboratory assessments included measurements of alanine transaminase (ALT) and aspartate transaminase (AST). Inclusion criteria comprised individuals aged 18 or older, while those with acute liver failure, decompensated cirrhosis, or hepatocellular carcinoma were excluded.

### 2.2. Vibration Controlled Transient Elastography Protocol

All VCTEs were completed by three experienced technicians (each with greater than 500 evaluations) within the outpatient hepatology office using a FibroScan 530 compact model (Echosens, Paris, France). Probe selection of either the M or XL probe was based on device recommendations after assessment of skin to liver capsule distance. All patients were fasting three hours before VCTE. Patients were in a supine position with their right arm elevated above the head. Each VCTE was completed at two separate ICS sites, each with the same probe, that were considered adequate by the VCTE device with a success rate greater than 60%. 10 or more adequate measurements were obtained at each ICS site with an interquartile range-to-median ratio (IQR/M) ≤ 30%. Liver stiffness measurements (LSM, kPa) were staged as follows: <5 kPa (stage 0), 5–7.9 kPa (stage 1), 8–9.9 kPa (stage 2), 10–13.9 kPa (stage 3), and 14+ kPa (stage 4) [11].

### 2.3. Comparison of Intercostal Spaces

VCTE results were reported as first ICS and second ICS. LSM results were directly compared for each participant’s median kPa and degree of variation between the two sites. Additionally, the frequency of each fibrosis stage (0–4) as suggested by the kPa range above was compared between both ICS sites.

Subjects were grouped into the following BMI classes: no obesity: BMI < 25, overweight: BMI 25–29.9, class 1 obesity: BMI 30 to 34.9, class 2 obesity: BMI 35–39.9, class 3 obesity: BMI 40 or higher. The variation between LSM at both ICS sites was compared based on ranges in kPa within each BMI class.

### 2.4. Statistical Analysis

We analyzed demographic and laboratory data both as continuous and categorical variables. Continuous variables were presented as means ± standard deviations (SD) or 95% confidence intervals (CI), while categorical variables were expressed as numbers or percentages. Categorical variables were assessed using Chi-square or Fisher’s exact test when appropriate, while an independent two-tailed t-test was employed for normally distributed continuous variables. ANOVA assessed average LSM values at two ICS sites across BMI classes. A *p*-value < 0.05 was considered statistically significant for all analyses. Statistical analyses were performed using SPSS software (SPSS Inc., Chicago, IL, USA, version 25.0 for Windows).

## 3. Results

### 3.1. Demographics

Adequate LSM values were successfully obtained at two ICS sites using the same probe in 1107 out of 1185 (93.4%) subjects. Table 1 displays the general demographics of the cohort. The cohort was primarily female (56.9%). Non-Hispanic white was the most common ethnicity at 85.2%, followed by black (9.7%) and Hispanic (5.1%). The average age was 54.3 (SD 13.9) years. A total of 71.4% of subjects had obesity, divided into class 1 (28.9%), 2 (21.3%), and 3 (21.2%). Average weight and BMI were 112.6 (SD 26.1) kg and 34.6 (SD 7.4) kg/m^2^, respectively. Metabolic co-morbidities included T2DM (41.6%), hypertension (57.3%), and dyslipidemia (49.7%). Average ALT U/L was 52.5 ± 45.2, and AST U/L was 43.4 ± 38.2. The average controlled attenuation parameter (CAP, dB/m), a measurement for the severity of steatosis, was similar at both the first 323 (SD 56.4) and second ICS sites 321 (SD 56.8), respectively. Average LSM (kPa) measurements were also similar at both ICS sites at 11.7 (SD 10.7) compared to 12.1 (SD 11.4), respectively. VCTE measurements suggested none to minimal liver fibrosis in 48.3%, moderate fibrosis in 10.7%, and advanced fibrosis in 40.9% of the cohort.

### 3.2. With-In Individual Variation of Liver Stiffness Measurements

**Figure 1:**
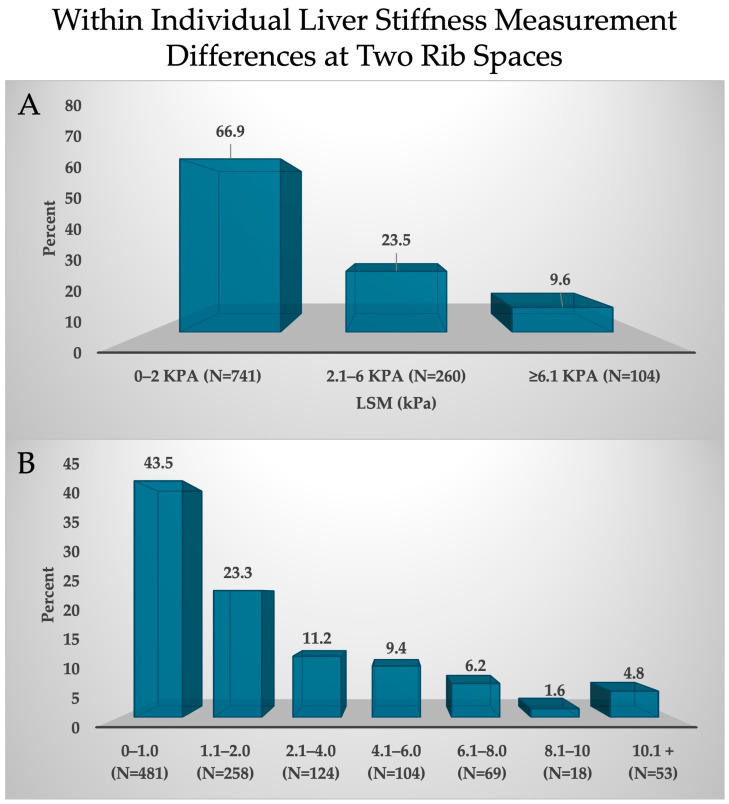
Variation of liver stiffness measurements in kPa at both intercostal spaces on an individual level. (**A**) represents differences among larger LSM groups and (**B**) shows differences among smaller groups. Abbreviations: LSM (liver stiffness measurement), kPa (kilopascals).

The variation of LSM values between the first and second ICS sites is shown in Figure 1A,B. Approximately 67% of the cohort exhibited a small difference within 2 kPa between the two ICS sites. Notably, 17.2% had discordant values of 2–4 kPa, 11.2% of subjects were noted to have 4.1–10 kPa differences, and were almost 5% greater than 10.1 kPa discordance at two ICS sites.

### 3.3. Within-Individual LSM Variation and BMI Classification

**Figure 2:**
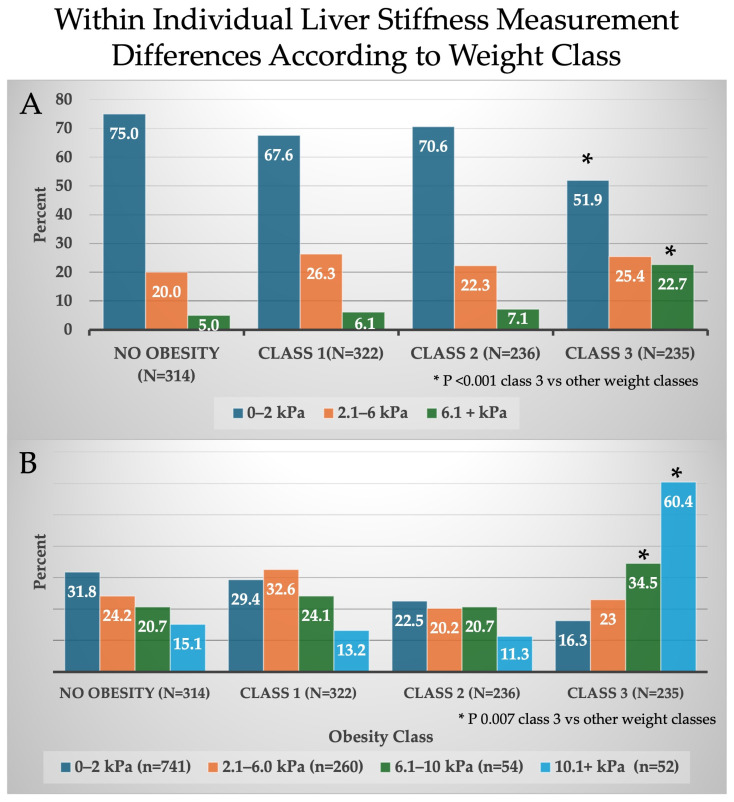
Overall range of variation in liver stiffness measurements (kPa) according to BMI class. (**A**) represents significantly higher variation in class 3 obesity compared to other BMI classes (* *p* < 0.001). (**B**) represents significant variation in class 3 obesity compared to other BMI classifications (* *p* = 0.01).

Figure 2A,B demonstrates LSM variation across BMI classes. The smallest variation between the two ICS sites (0–2 kPa) was noted in 75% of subjects without obesity, 67.6% in class 1 obese subjects, and 70.6% in class 2 obese subjects. However, a significantly lower prevalence of 51.9% of 0–2 kPa variation was noted in those with class 3 obesity (*p* < 0.001). Subjects with 6.1 kPa or greater differences between ICS sites was noted in class 3 obese subjects (22.7%), a prevalence three to four times greater than all other BMI classes (*p* < 0.001). Class 3 obese individuals had the highest degree of kPa differences (6.1–10 kPa) and (10+ kPa) at 35% and 60%, respectively (*p* = 0.007).

### 3.4. Comparison of Group Averages of LSM Between Intercostal Spaces

**Figure 3:**
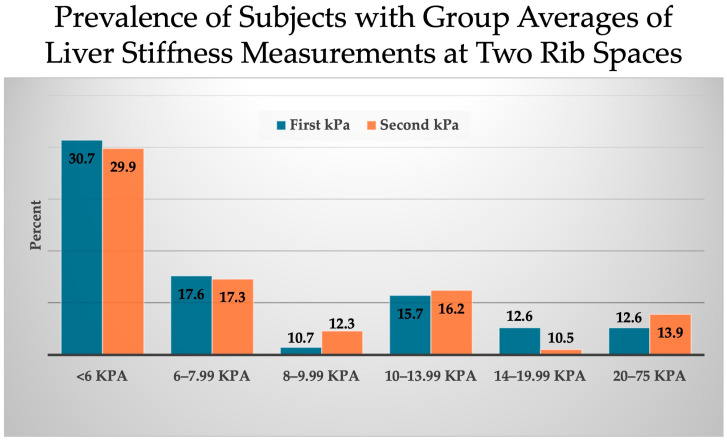
Average liver stiffness measurement at both first and second intercostal space by kPa range. Abbreviations: kPa (kilopascals).

The group averages of LSM differences between the two ICS sites demonstrate very similar values (Figure 3). A total of 30.7% of the cohort had a LSM measurement at the first ICS as <6 kPa, compared to 29.9% at the second ICS. In total, 28.3% of subjects had 6–10 kPa at site one compared to 29.6% at rib site 2. LSM values between 10–20 kPa occurred at site 1 in 28.3% and site 2 in 26.7%. Both ICS sites had a similar prevalence (12.6% compared to 13.9%) of subjects with LSM values equal to or greater than 20 kPa.

**Figure 4:**
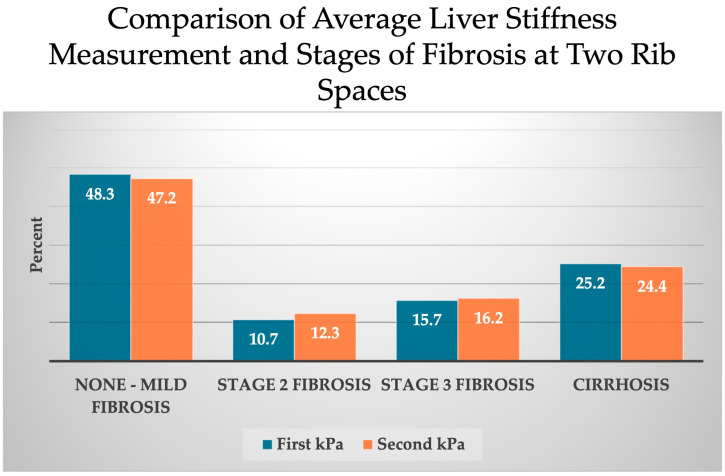
Liver stiffness measurements grouped by fibrosis stage for both first and second intercostal space. Abbreviations: kPa (kilopascals).

Figure 4 demonstrates no significant differences between the two ICS sites regarding the suggested stages of fibrosis. None to mild fibrosis (F0–1) was identified in 48.3% of subjects at the first ICS and 47.2% at the second ICS. Stage 2 fibrosis was present in 10.7% of subjects at the first ICS and 12.3% at the second ICS. Stage 3 fibrosis was identified in 15.7% of first ICS and 16.2% of second ICS. Cirrhosis was suggested in 25.2% of subjects at the first ICS and 24.4% at the second ICS. When the group averages of LSM were categorized by fibrosis stage, there was no difference between the first and second ICS sites in 65.7% of participants, one stage difference in 30.4%, and two to four stages different in 4.0% of subjects.

### 3.5. Intercostal Space Discordance and BMI Classification

**Figure 5:**
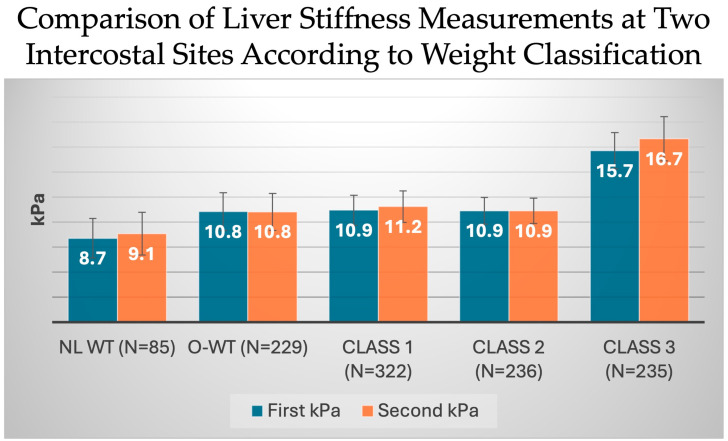
Elastography results demonstrating average kPa variation in liver stiffness measurements between intercostal spaces. *p* < 0.001 class 3 LSM compared to all other BMI classes. Abbreviations: kPa (kilopascals), NL Wt (normal weight), O-WT (overweight).

Subjects with class 3 obesity had significantly higher LSM at both the first and second ICS sites compared to individuals in other BMI classes (Figure 5). Of the total cohort, 85 (7.7%) subjects were classified as normal weight, while 229 (20.7%) subjects were overweight. At the first and second ICS sites, LSM values were recorded as 8.7 and 9.1 kPa, respectively, in subjects of normal weight, and 10.8 kPa for both ICS sites in those who were overweight. In the obese individuals, 29.1% (n = 329) were classified as having class 1 obesity, and class 2 and 3 obese classifications were noted in 21.3% (n = 236) and 21.2% (n = 235), respectively. In subjects with class 3 obesity, the average LSM was statistically greater at the first and second ICS sites 15.7 and 16.7 kPa, respectively, compared to 8.7 and 9.1 kPa in patients without obesity, 10.8 and 10.8 kPa in overweight subjects, 10.9 and 11.2 kPa in those with class 1 obesity, and 10.9 and 10.9 kPa in those with class 2 obesity (*p* < 0.001 avg LSM in class 3 vs. all other BMI classes.

## 4. Discussion

This is the first study to determine the concordance of VCTE at two ICS sites obtained in a single session across all weight classifications. Overall, 742 (33%) subjects had greater than two kPa differences at two ICS sites. A total of 105 (10%) individuals had more than six kPa variances most notably in subjects with a BMI ≥ 40 kg/m^2^. This degree of within-individual kPa difference is clinically significant when considering serial measurements after weight-loss and/or to determine the effectiveness of liver-directed medical therapy. When comparing the average liver stiffness values at two rib sites for the entire cohort, the suggested stage of fibrosis was the same in 66% of individuals, 30% were within one stage and only 4% of cases were discordant by more than two stages. Taken together, a single ICS measurement may be adequate to assess the overall stage of liver fibrosis for a group of individuals, but the significant within-individual variation demonstrated at two sites may be insufficient to assess the improvement of liver fibrosis from either weight-loss or liver-directed medical treatment.

Previous investigations that evaluated VCTE in multiple rib spaces focused on group-averages of liver stiffness. Boursier and colleagues evaluated 46 subjects, mostly with alcohol and viral hepatitis, identifying that a high BMI and rib space decreased the group-average LSM agreement but did not evaluate the within-individual variance at multiple rib sites [7]. A study in 91 Asian patients with chronic hepatitis B virus performed a liver biopsy and VCTE measurement at the fifth, sixth, seventh, or eighth rib space; no significant difference in the stage of fibrosis was identified [8]. Song and colleagues compared liver stiffness elastography using GE LOGIQ E9 to MRE in 47 subjects (only 8 with MASH) showing that the seventh and eighth ribs spaces, but not the nineth, had a good correlation (0.68–0.76) with MRE [9]. Lastly, the average VCTE measurement from two intercostal spaces in 16 hepatitis C patients was compared to seven different elastography machines showing moderate concordance [10].

Although liver biopsy is the gold standard to diagnose MASH and the stage of fibrosis, it cannot be utilized as a screening test due to its invasive, costly, and potential procedural complications that include infection and bleeding. Additionally, liver biopsy is impractical as a screening tool due to the 5% prevalence of high-risk MASH with stages 3–4 fibrosis demonstrated by VCTE screening in the USA [12]. Due to cost, general availability, and office base point-of-care options, VCTE will likely become the most common NIT used to identify MASH subjects with liver fibrosis that are appropriate for medical therapy. Subjects will also have to undergo serial measurements with VCTE to determine if the stage of liver fibrosis improves with medical therapy.

The accuracy of VCTE can be limited dependent upon body habitus. A BMI ≥ 40 kg/m^2^ has been associated with an increase in unreliable liver stiffness values results due to increased skin-to-liver capsule distance [13,14]. Caussy et al. reported a 65% discordance of two or more fibrosis stages between VCTE and liver biopsy in subjects with class 3 obesity [15]. The decrease in reliability can be attributed to technical challenges related to probe placement and signal attenuation caused by increased tissue thickness in individuals with higher BMI. We speculate the significant within-individual variation noted in our study at two ICS sites, which was particularly predominant in class 3 obese individuals, is also likely attributed to truncal obesity and signal reduction from skin-to-liver capsule.

Resmetirom, a THR-beta agonist, has been FDA-approved as a liver-directed medication for subjects with MASLD and stages 2–3 fibrosis [16]. It acts on the hepatic thyroid receptor, which regulates multiple metabolic cellular pathways. To date, this is the only medication approved for MASH demonstrating 10–12% of subjects with a one-stage decrease in liver fibrosis. Phase 2 trials with liver-directed mechanisms (Efruxifermin, Pegozafermin) that target FGF-21 or weight-loss combinations of GLP-1/GIP/Glucagon (Tirzepatide, Survodutide) have shown improvement in liver fibrosis and are currently in phase 3 investigations [17,18,19,20]. Importantly, a liver biopsy was used to determine efficacy whereas, going forward the effectiveness from medical or weight-loss therapy will be evaluated using noninvasive tests.

Recently, expert guidance has been published proposing a VCTE 10–19.9 kPa range to identify individuals with MASH and significant fibrosis that warrant Resmetirom therapy [21]. If liver stiffness on VCTE is greater than or equal to 20.1 kPa then liver-directed medical therapy is not advised unless a liver biopsy reveals stages 2 or 3 fibrosis due to the concern for cirrhosis. Due to the potential overestimation of liver stiffness in subjects with class 3 obesity, a second noninvasive measure of liver fibrosis, either the ELF blood test or MRE can be considered in those with a VCTE of 20 kPa or greater to determine if a liver biopsy is warranted. Advanced liver fibrosis strongly correlates with adverse liver-related outcomes and mortality [22]. Agile 3+ and Agile 4 scores are derived from VCTE liver stiffness values including clinical and laboratory data: AST, ALT, platelets, diabetes, age, and gender. These formulas have improved upon the negative and positive predictive values for advanced fibrosis and cirrhosis, respectively, as well as predicting poor liver-related outcomes and are easy to calculate with the FibroScan (Echosens) app [23].

In the phase 3 study demonstrating resmetirom’s efficacy, the average LSM had a decrease of 2.5 to 3.3 kPa that was associated with ≥1-stage fibrosis improvement on liver biopsy [24]. A 20–30% decrease in liver stiffness may predict medication effectiveness [25,26]. Using 10 kPa as the cut-off guideline to initiate liver-directed therapy, a 2–3 kPa difference from the baseline would meet the defined effective criteria for continuation of therapy. Our results show that 33% of all BMI classes revealed ≥ 2 kPa, and 10% of individuals with class 3 obesity had >6 kPa within-individual variation at two ICS sites thereby concluding a false positive medical outcome. Additionally, VCTE overestimation of LSM in class 3 obese individuals can lead to a dramatic decrease (23 kPa to 8 kPa) after significant weight-loss, thereby adding to a false improvement in liver fibrosis [27]. This degree of variation will have significant implications on clinicians, patients, and healthcare insurance plans to assess patients’ effectiveness from resmetirom. Future research is warranted to determine if obtaining two baseline LSM values from two intercostal spaces and investigating if either the lowest or average values pre and post therapy provide a better prediction for medical effectiveness.

Limitations include lack of randomization, potential confounding factors, and selection bias that are inherent in a retrospective study. This was a single-center experience; however, our three VCTE technicians have years of experience. Since three technicians performed the scans there is potential for inter-operator variability; however, we feel our center’s experience is generalizable to a community healthcare setting. We used the FibroScan 530 compact model and a recent SmartExam upgrade by Echosens may improve within-individual variation in obesity due to a 28% increase in skin-capsule-distance; however, this has yet to be investigated [28].

## 5. Conclusions

To conclude, significant within-individual variation in LSM occurs across all BMI classes and is particularly greatest in class 3 obese individuals. Further research is warranted to address clinical challenges using serial VCTE to ensure accurate effectiveness from liver-directed medical therapy or weight-loss.

## Figures and Tables

**Table 1:** General demographics for entire cohort. Variables are expressed as mean (SD) or n (%). The fibrosis stage is based on lowest reading between both intercostal spaces. Abbreviations: ALT (alanine transaminase), AST (Aspartate transaminase), BMI (body mass index), VCTE (vibration controlled transient elastography). 3.1 Within individual variation of liver stiffness measurements.

N = 1107	Overall
Gender Female n, (%)	630 (56.9)
Age, years	54.3 (13.9)
Weight, kg	112.6 (26.1)
BMI, kg/m^2^	34.6 (7.4)
Race (n %)	
Non-Hispanic White	943 (85.2)
Black	107 (9.3)
Hispanic	56 (5.1)
First CAP, dB/m	323 (56.4)
Second CAP, dB/m	321.7 (56.8)
First LSM, kPa	11.7 (10.7)
Second LSM, kPa	12.1 (11.4)
ALT, IU/L	52.5 (45.3)
AST, IU/L	43.4 (38.2)
Metabolic Factors n (%)	
Type 2 Diabetes Mellitus	461 (41.6)
Hypertension	634 (57.3)
Dyslipidemia	550 (49.7)
Obesity Class n (%)	
Normal Body Weight	85 (7.7)
Overweight	229 (20.7)
Class 1	322 (29.1)
Class 2	236 (21.3)
Class 3	235 (21.2)
Fibrosis Stage by VCTE n (%)	
0–1	535 (48.3)
2	119 (10.7)
3	174 (15.7)
4	279 (25.2)

## Data Availability

The data are not publicly available due but may be released at the authors discretion upon reasonable request.
